# Supplementary material for: Coral high molecular weight carbohydrates support opportunistic microbes in bacterioplankton from an algae-dominated reef
Source: mSystems. 2024 Oct 22;9(11):e00832-24. doi: 10.1128/msystems.00832-24 (PMC11575353; doi:10.1128/msystems.00832-24)
Supplement: Supplemental Material — Supplemental methods, Fig. S1–S7, and Tables S1–S8. [file msystems.00832-24-s0001.pdf]

Coral high molecular weight carbohydrates support opportunistic microbes in bacterioplankton from an algae-dominated reef

Bianca M. Thobor[a#], Andreas Haas[b], Christian Wild[a], Craig E. Nelson[c], Linda Wegley Kelly[d], Jan-Hendrik Hehemann[e,f], Milou G. I. Arts[b], Meine Boer[b], Hagen Buck-Wiese[e,f], Nguyen P. Nguyen[e,f], Inga Hellige[e,f] and Benjamin Mueller[a,g,h]

[a]Department of Marine Ecology, University of Bremen, Bremen, Germany
[b]Department of Microbiology & Biogeochemistry, NIOZ Royal Netherlands Institute for Sea Research, Texel, The Netherlands
[c]Daniel K. Inouye Center for Microbial Oceanography: Research and Education, Department of Oceanography and Sea Grant College Program, University of Hawaiʻi at Mānoa, Honolulu, Hawaiʻi, USA
[d]Marine Biology Research Division, Scripps Institution of Oceanography, University of California, San Diego, California, USA
[e]MARUM Centre for Marine Environmental Sciences, University of Bremen, Bremen, Germany
[f]Department of Marine Glycobiology, Max Planck Institute for Marine Microbiology, Bremen, Germany
[g]Department of Freshwater and Marine Ecology, University of Amsterdam, Amsterdam, the Netherlands
[h]CARMABI Foundation, Willemstad, Curaçao

#Corresponding author: thobor@uni-bremen.de

Supplementary methods

Carbohydrate microarray analysis

Macroalgae tissue extracts were analysed using carbohydrate microarrays, as described by Vidal-Melgosa et al. (1). Shortly, AIR-washed tissue powder was sequentially extracted using autoclaved UW, 50 mM EDTA pH 7.5, and 4 M NaOH with 0.1% w/v $NaBH_4$ and stored at -20°C until further processing. UW- and EDTA extracts were additionally diluted by a factor of 1:2 to adjust viscosity. Extracts were vortexed (5 sec), centrifuged (10 min at 16,000 × *g*), and transferred into 384-microwell plates for printing of microarrays. Printing was done in four concentrations (1:2, 1:10, 1:20 and 1:40) per extract, diluted with printing buffer (ArrayJet, Roslin, UK), and printed using a microarray robot (Sprint, ArrayJet, Roslin, UK) on nitrocellulose membrane (0.45 µm, Whatmann). All solvents were also printed on the microarrays and did not result in unspecific signals. Printed microarrays were first incubated for 1 h in phosphate buffered saline (PBS, 1x) with 5% (w/v) non-fat milk powder (MPBS), and then for 2 h with monoclonal antibodies (mAbs) in MPBS which bind specifically to epitopes present in sulphated fucan (i.e., BAM1, BAM2, BAM3 and BAM4). Microarrays were then washed in PBS, incubated for 2 h with secondary control antibodies (anti-rat, anti-mouse and anti-His conjugated to alkaline phosphatase, all not resulting in any signals) in MPBS and afterwards washed in deionised water. Microarrays were developed in a solution of 5-bromo-4-chloro-3-indolylphosphate and nitro blue tetrazolium in alkaline phosphatase buffer (100 mM NaCl, 5 mM MgCl2, 100 mM Tris-HCl, pH 9.5) and probe binding signal intensity was analysed using the software Array-Pro Analyzer 6.3 (Media Cybernetics). The highest signal intensity of the dataset (which included 27 other mAbs and carbohydrate binding modules (CBMs)) was set to 100, all other signals were adjusted accordingly, and low arbitrary units were removed by applying a cut-off of 5. Binding intensities cannot be compared between species, nor between mAbs, because binding efficiencies may differ.

Fucoidan extraction from biomass

Up to 2 g of dried algae biomass powder (i.e., oven-dried *Lobophora* and *Dictyota* thalli) were weighed and resuspended in a final concentration of 25 mM EDTA (pH= 8) in 1 L of ultrapure water (UW). The suspension was autoclaved at 123°C for 25 min and afterwards filtered over a glass fibre filter (0.7 µm) and cellulose nitrate filter (0.45 µm) prior to polyvinylpolypyrrolidon (PVPP, Merck/Sigma Aldrich) treatment. To bind polyphenols, 1 L of sample was mixed with 50 g of PVPP and 1 M phosphate buffer at a final concentration of 50 mM (pH= 6), stirred for 1 hour at room temperature and filtered again over a glass fibre filter. 10 µL of alginate lyase (Sigma) was added to the solution and incubated at 37°C for 24 h. The filtrate was run over a 30 kDa Biomax ultrafiltration membrane (Merck Millipore) using an

Amicon filtration device, washed 3 times against UW, resuspended in 200 mL UW and mixed with 1M Tris-HCl, pH 7.5 at a final concentration of 50 mM. The sample was passed through an anion exchange column, which had been conditioned with 2 column volumes of 0.5 M NaCl and 20 mM Tris-HCl at 5 mL min^{-1} flow rate, 2 column volumes of 5 M NaCl and 20 mM Tris-HCl at 5 mL min^{-1} flow rate and again 2 column volumes of 0.5 M NaCl and 20 mM Tris-HCl at 5 mL min^{-1} flow rate. The sample was loaded to the column and washed with 3 column volumes of 0.5 M NaCl and 20 mM Tris-HCl prior to eluting fucoidan in 5 M NaCl and 20 mM Tris-HCl at a 5 mL min^{-1} flow rate. The sample was dialysed against three times UW and concentrated using a 30 kDa ultrafiltration membrane with an Amicon filtration device. The final solution was freeze dried to obtain fucoidan powder. From the fucoidan powder, 5 mg L^{-1} solutions were prepared in UW. 500 µL of these stocks were added to 500 µL of 2 M HCl in pre-combusted glass ampoules (450°C, 4.5 h), acid-hydrolysed at 100°C for 24 h, dried in an acid-resistant vacuum concentrator, re-suspended in 1 mL of UW, and further processed for HPAEC-PAD analyses (see main methods section).

Supplementary Figures

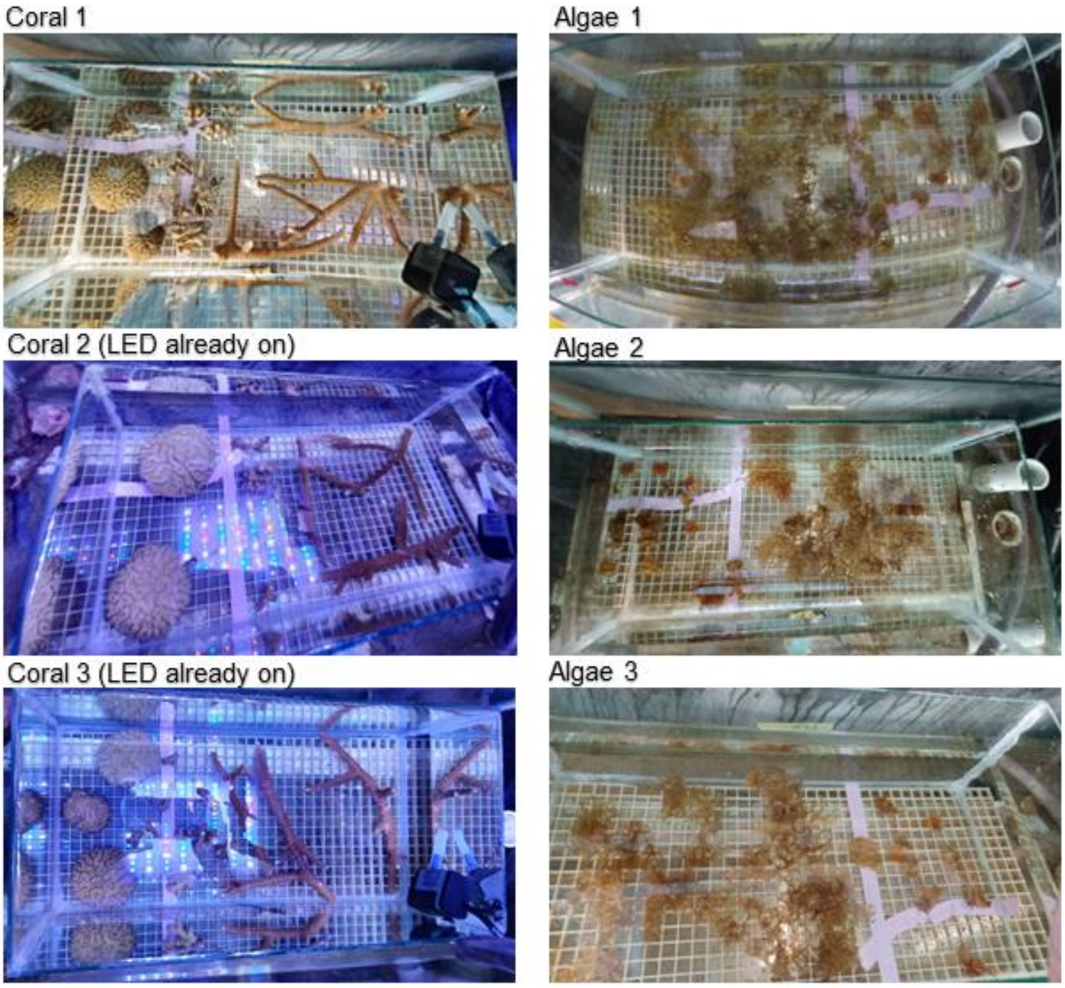

Figure S1. Pictures taken at the start of the exudate incubations with corals (left) and macroalgae (right). Mean relative cover of the aquarium floor was 34 % (± 5 sd) for coral- and 50% (± 14 sd) for macroalgae incubations (analyzed with ImageJ).

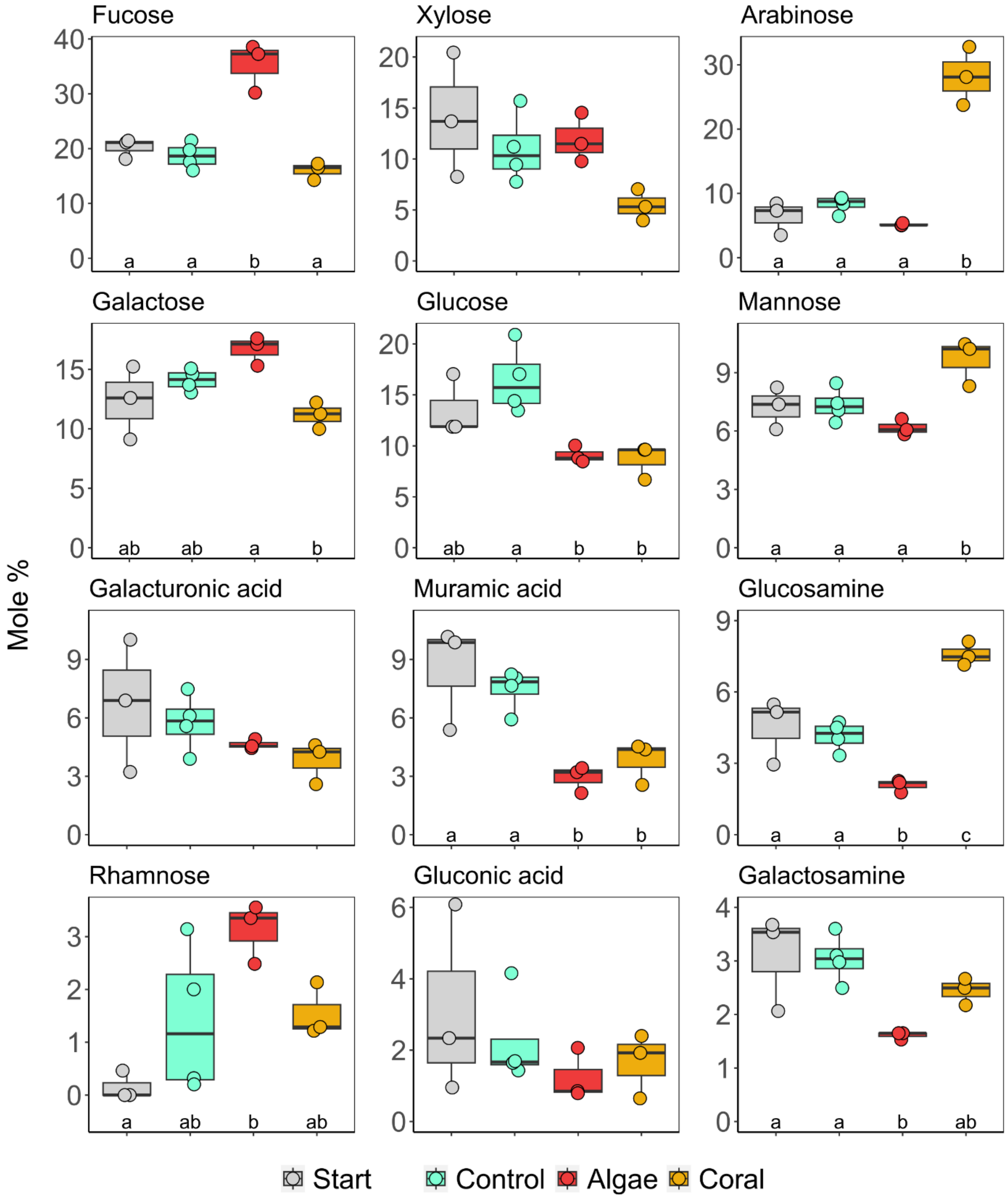

Figure S2. Carbohydrates released by corals and macroalgae in mole %. Boxes represent median ± 95% confidence intervals, and points represent replicates. Letters indicate significant differences between treatments (HSD test). Note different scales of vertical axes. See ANOVA results in Table 2. Raw data to this figure is available in Supplementary Table S4.

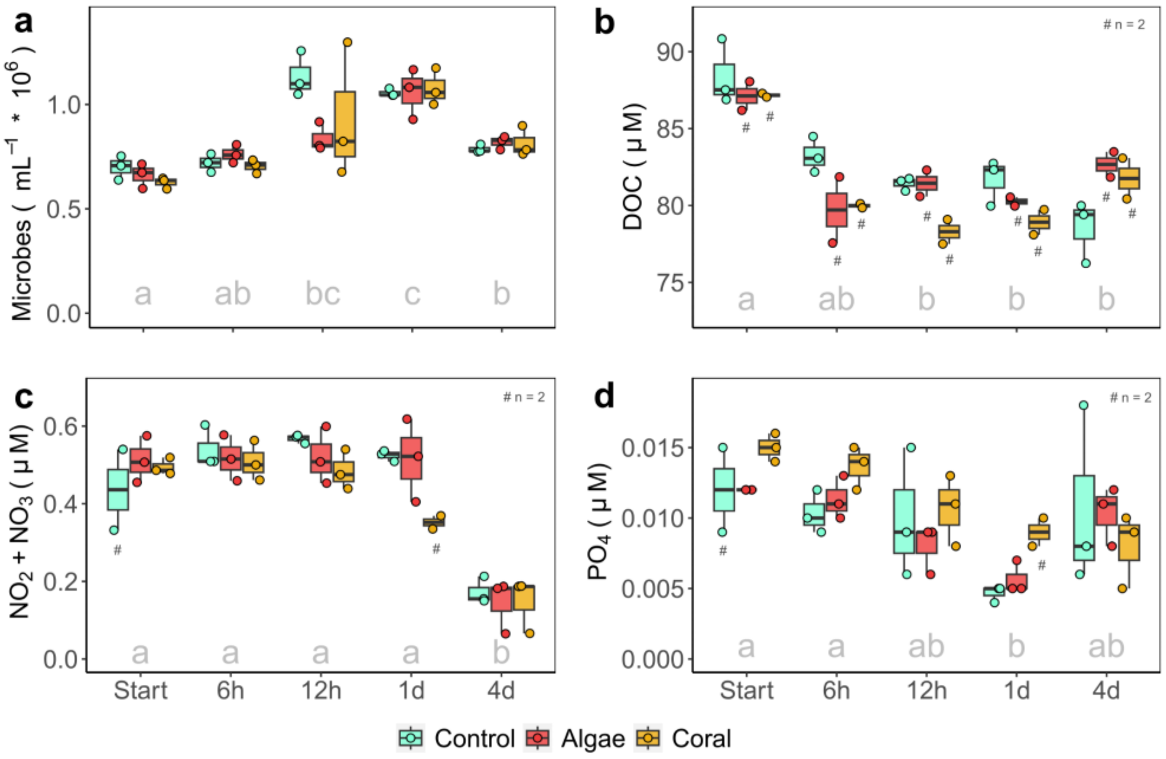

Figure S3. Development of (**a**) microbial cell densities, and (**b-d**) dissolved nutrient concentrations during dark incubations with corals and macroalgae HMW exudates and background HMW DOM (controls). DOC = Dissolved organic carbon, NO2 + NO3 = combined dissolved nitrite and nitrate, PO4 = dissolved phosphate. Different letters indicate significant differences between times ($p < 0.05$, pairwise t-tests (**a**) or Dunn's tests (**b-d**), Bonferroni adjusted). Hashtags (in **b-d**) indicate groups where one sample had to be excluded due to sampling or measurement errors (i.e., n = 2). Raw data to this figure is available in Supplementary Table S6.

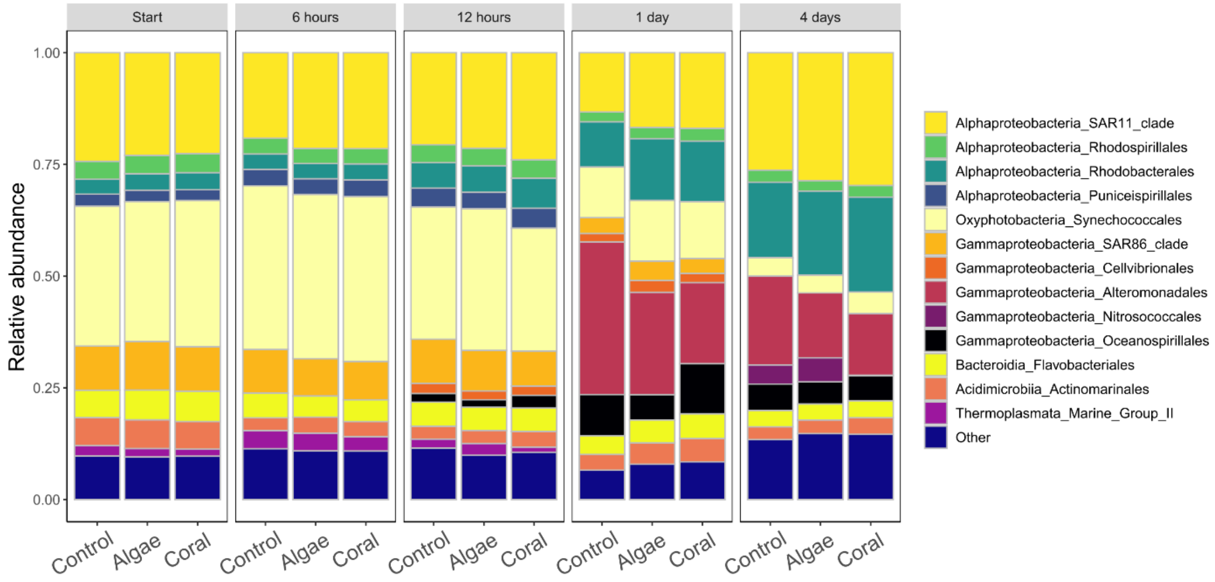

Figure S4. Mean relative abundance of dominant orders (> 2% mean relative abundance in any treatment per timepoint) during bacterioplankton incubations with HMW DOM from control- macroalgae- and coral-incubations.

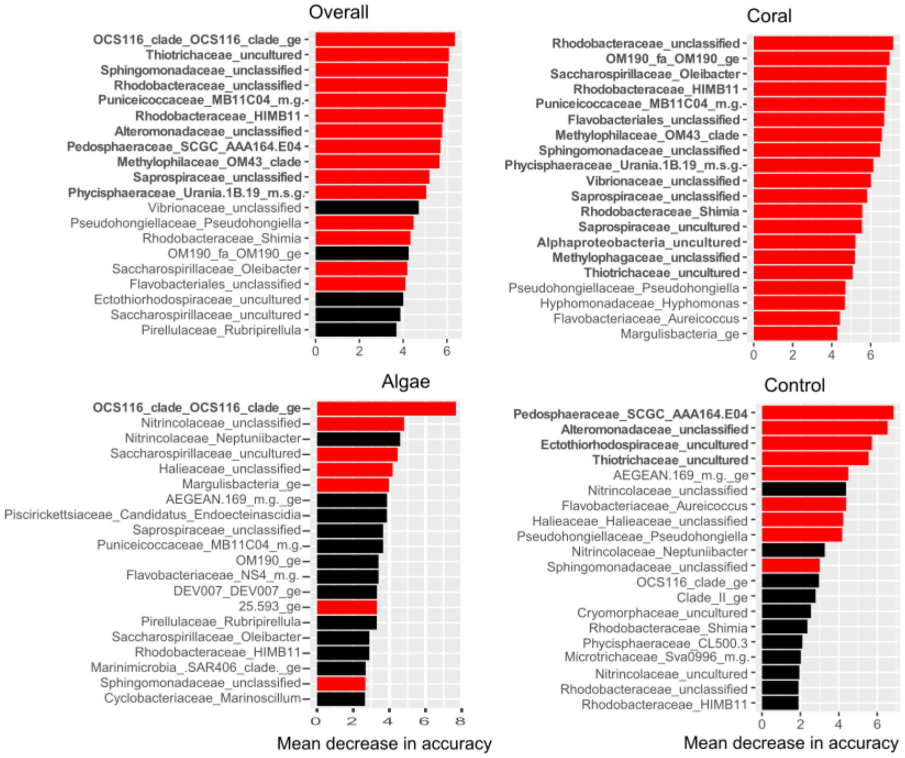

Figure S5. RFpermute variable importance plots of the bacterioplankton community composition after four days of bacterioplankton incubation. A high mean decrease in accuracy (MDA) of the model when a specific variable (genus) is removed equals a high importance in classification of the overall model, coral, algae, and control samples. Red bars indicate significant ($p < 0.05$) contribution to permutational random forest classification. Bold genera were significant and had an MDA score > 5.

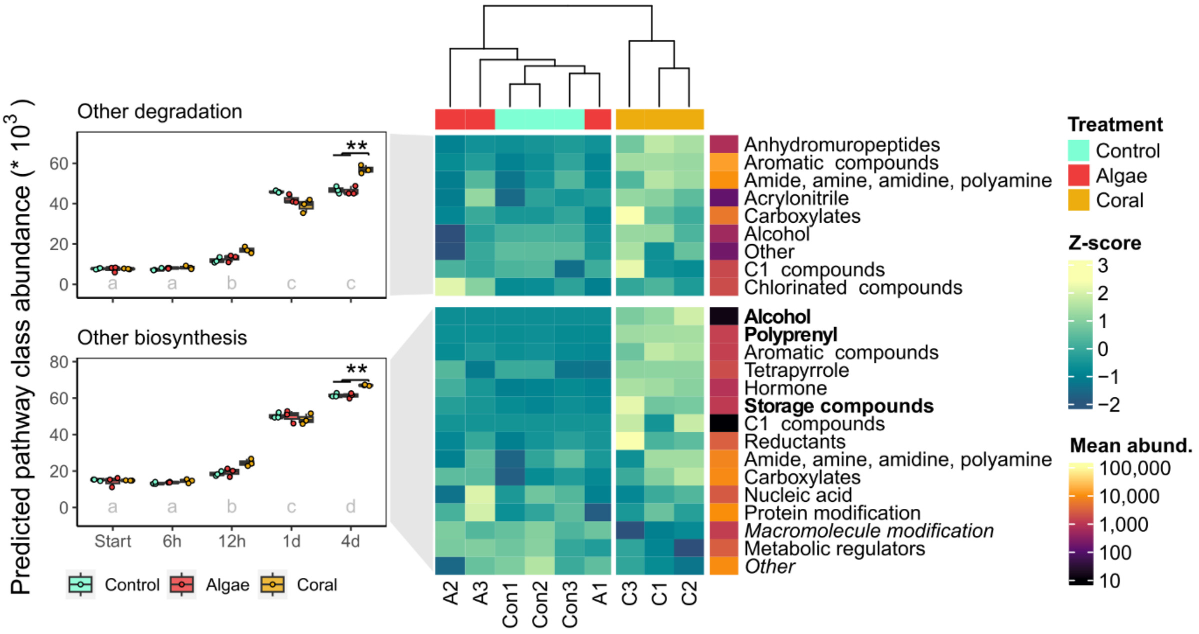

Figure S6. (**a**) Development of other predicted metabolic degradation and biosynthesis pathways during bacterioplankton incubations with algae and coral HMW DOM and background HMW DOM (controls). Different letters indicate significant differences between times ($p < 0.05$, pairwise t-tests, Bonferroni adjusted). Asterisks indicate significant differences between treatments (* $p < 0.05$, *** $p < 0.001$, **** $p < 0.0001$, pairwise t-tests,

Bonferroni adjusted). Predictions are based on the MicFunPred analysis tool, using the MetaCyc database. (**b**) Predicted pathway abundance by type as Z-scores after four days of dark incubation. The dendrogram on the top represents the Euclidean distance between samples including all pathway types of all classes. SYN = biosynthesis, DEG = degradation. Bold pathway types in (**b**) indicate a significant increase in coral treatments compared to controls (fdr-corrected $p < 0.001$, log2 fold change > 0.5, DESeq2 on all pathway types), and italicized types a significant decrease.

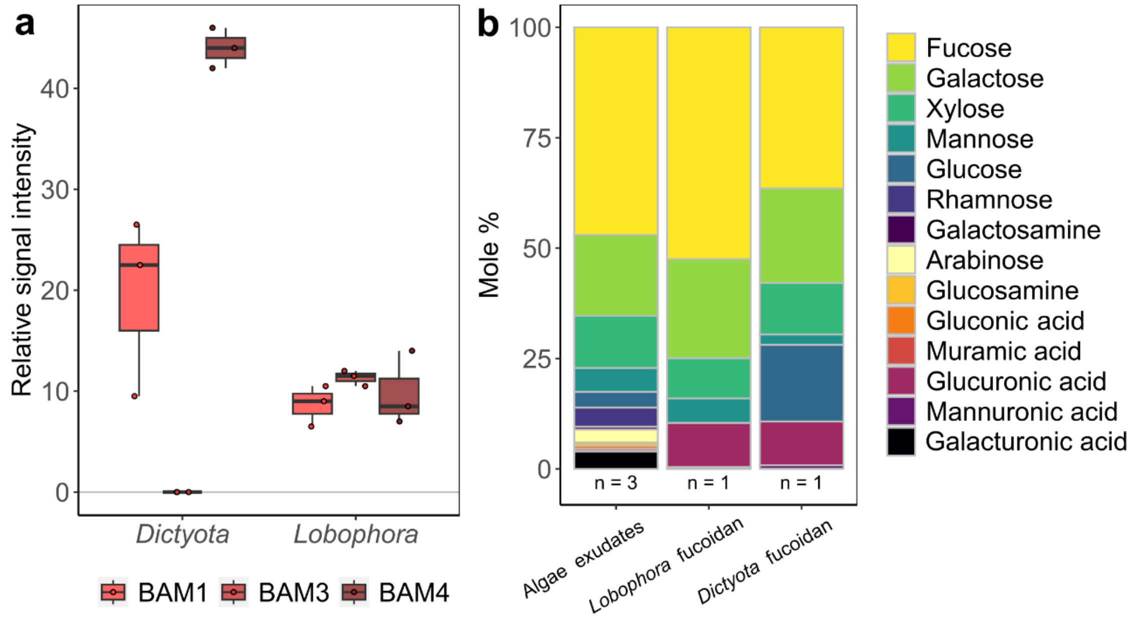

Figure S7. Evidence for fucoidan contribution to brown macroalgae HMW DOM. (**a**) Relative signal intensity of monoclonal antibodies binding to epitopes present in sulphated fucan for macroalgae tissue extracts of *Dictyota* sp. and *Lobophora* sp. (sum of signals from UW, EDTA, and NaOH extracts). BAM2 was not detected in any sample. (**b**) Hydrolysable monosaccharide composition of algae exudates (control-corrected) and fucoidan extracted from dried tissue of macroalgae used in the present study. Relative signal intensity (**a**) cannot be compared among monoclonal antibodies, nor between algae species, because of different binding efficiencies.

Supplementary Tables

Table S1. RFpermute model confusion matrix for classification of bacterioplankton communities (genus-level) after four days. LCI_0.95 / UCI_0.95 = lower / upper 95% confidence interval. RF models are based on 5,000 trees each, with 16 variables tried at each split and 100 permutational replicates.

	**Algae**	**Control**	**Coral**	**percent correct**	**LCI_0.95**	**UCI_0.95**
Algae	0	3	0	0.0	0.0	70.8
Control	3	0	0	0.0	0.0	70.8
Coral	0	0	3	**100.0**	29.2	100.0
Overall	NA	NA	NA	33.3	7.5	70.1

Table S2. Microbial orders significantly enriched with coral exudates in the present study, and their previously described associations with coral mucus, coral exudates, coral disease and stress, and algae exudates.

	**Order**	**Found in coral mucus**	**Increase in seawater with coral mucus / exudates**	**Increase with coral disease / stress**	**Increase in seawater with algae exudates**
Alpha-proteobacteria	**Rhodobacterales**	(2–5)	(6–9)	(10–12)	(6)
Gammaproteo-bacteria	**Vibrionales**	(5, 13)	(7, 8, 14)	(11, 12, 15, 16)	(6)
	**Thiotrichales**	(4)		(17)	
Bacteroidetes	**Flavobacteriales**	(2–4)	(7, 8)	(11, 12, 15)	(6)
	**Saprospirales**			(18)	
Planktomycetes	**OM190**		(6)		
	**Phycisphaerales**			(19, 20)	

Table S3. Raw data to Fig. 2.

		Fig. 2a	Fig. 2b	Fig. 2c	Fig. 2d
**Name**	**Treatment**	**DOC (uM)**	**HMW DOC (µM)**	**HMW DOC as CH (µM)**	**HMW DOC as CH (%)**
A1	Start	69.84	NA	NA	NA
A2	Start (1)	71.36	1.60	0.39	24.42
A3	Start	73.85	NA	NA	NA
C1	Start	72.03	NA	NA	NA
C2	Start	73.62	NA	NA	NA
C3	Start (2)	70.70	1.98	0.57	25.55
Con1	Start (3)	69.36	2.31	1.10	47.51
Con2	Start	74.08	NA	NA	NA
Con3	Start	75.57	NA	NA	NA
Con4	Start	69.16	NA	NA	NA
A1	Algae	84.85	2.28	1.75	76.71
A2	Algae	80.03	2.33	1.78	76.53
A3	Algae	79.81	3.65	2.50	68.56
C1	Coral	85.78	3.34	2.64	79.01
C2	Coral	79.74	2.71	1.38	70.71
C3	Coral	73.39	2.38	1.19	49.78
Con1	Control	76.58	2.82	0.99	35.23
Con2	Control	76.73	1.95	0.67	34.52
Con3	Control	75.91	1.87	0.66	35.41
Con4	Control	72.28	NA	0.87	NA

Table S4. Raw data for Fig. 3 & S2. Fuc = fucose, Rha = rhamnose, GalN = galactosamine, Ara = arabinose, GlcN = glucosamine, Gal = galactose, Glc = glucose, Man = mannose, Xyl = xylose, GlcA = gluconic acid, MurA = muramic acid, GalA = galacturonis acid.

Time	Name	Treatment	Fuc	Rha	GalN	Ara	GlcN	Gal	Glc	Man	Xyl	GlcA	MurA	GalA
**Fig. 3 (µg L^{-1})**														
Start	S1	Start	2.19	0.00	0.40	0.33	0.62	1.43	1.35	0.84	0.78	0.75	1.61	1.23
Start	S2	Start	2.80	0.00	0.62	1.19	0.87	2.58	2.01	1.03	1.93	0.18	2.34	1.26
Start	S3	Start	6.56	0.14	0.69	2.05	0.98	3.06	5.72	2.77	5.72	0.85	2.52	1.17
End	A1	Algae	18.74	1.63	0.81	2.24	0.94	9.13	4.69	3.53	4.34	0.50	1.59	2.56
End	A2	Algae	18.37	1.22	0.89	2.28	1.22	8.28	4.58	3.15	5.18	1.22	2.42	2.86
End	A3	Algae	21.01	2.47	1.25	3.43	1.66	13.45	7.66	4.63	9.25	0.66	3.64	3.74
End	C1	Coral	12.58	1.63	1.81	22.82	6.75	8.35	5.58	8.74	3.70	0.59	2.98	2.33
End	C2	Coral	5.56	0.47	1.06	10.02	3.18	4.82	4.12	4.37	1.42	1.12	2.61	2.12
End	C3	Coral	5.77	0.43	0.97	7.25	2.60	4.48	3.53	3.05	2.15	0.77	2.32	1.68
End	Con1	Control	4.83	0.09	0.75	2.09	1.00	3.93	6.30	2.13	3.94	0.47	2.49	1.26
End	Con2	Control	3.91	0.36	0.72	1.53	0.94	2.92	2.69	1.49	1.29	0.36	2.24	1.32
End	Con3	Control	2.86	0.56	0.61	1.06	0.79	2.96	2.83	1.66	1.55	0.89	2.25	1.58
End	Con4	Control	4.67	0.05	0.77	2.01	1.16	3.56	4.43	1.67	2.42	0.48	2.77	1.56
**Fig. S2 (Mole %)**														
Start	S1	Start	28.64	0.00	4.80	4.75	7.42	17.09	16.10	9.99	11.20	8.25	13.79	13.59
Start	S2	Start	22.01	0.00	4.47	10.25	6.27	18.52	14.44	7.40	16.64	1.16	12.01	8.38
Start	S3	Start	24.08	0.52	2.32	8.21	3.31	10.23	19.13	9.25	22.95	2.62	6.04	3.62
End	A1	Algae	41.66	3.62	1.65	5.44	1.91	18.50	9.50	7.15	10.56	0.92	2.31	4.80
End	A2	Algae	41.49	2.77	1.84	5.63	2.52	17.04	9.43	6.49	12.79	2.30	3.58	5.47
End	A3	Algae	33.10	3.89	1.81	5.92	2.40	19.30	11.00	6.65	15.94	0.87	3.75	4.98
End	C1	Coral	17.55	2.27	2.31	34.80	8.62	10.61	7.10	11.11	5.64	0.69	2.71	2.75
End	C2	Coral	16.08	1.37	2.82	31.71	8.44	12.71	10.86	11.53	4.48	2.71	4.94	5.19
End	C3	Coral	19.33	1.44	2.99	26.59	8.00	13.68	10.79	9.30	7.88	2.16	5.07	4.76
End	Con1	Control	19.80	0.36	2.81	9.38	3.75	14.70	23.54	7.96	17.69	1.61	6.67	4.39
End	Con2	Control	25.48	2.38	4.28	10.88	5.61	17.33	16.00	8.83	9.22	1.95	9.56	7.25
End	Con3	Control	19.98	3.92	3.87	8.07	5.02	18.82	17.98	10.56	11.79	5.19	10.28	9.34
End	Con4	Control	23.20	0.24	3.50	10.93	5.29	16.10	20.02	7.57	13.16	1.99	9.00	6.56

Table S5. Raw data for Fig. 4. Values in mole % of algae tissue extracts, coral mucus, control-corrected fluxes (exudates), or ambient reef water.

Name	Treatment	Fucose	Rhamnose	Galactosamine	Arabinose	Glucosamine	Galactose	Glucose	Mannose	Xylose
Lob1	Lobophora tissue	33.86	0.63	0.15	0.00	1.68	15.35	12.56	10.73	10.61
Lob2	Lobophora tissue	31.60	0.41	0.03	0.00	1.76	16.74	18.72	9.88	9.44
Lob3	Lobophora tissue	34.97	1.03	0.17	0.00	2.08	19.13	7.50	10.93	11.91
Dic1	Dictyota tissue	23.31	1.29	0.00	0.00	1.10	14.55	14.11	4.53	7.56
Dic2	Dictyota tissue	17.12	0.37	0.00	0.00	0.83	16.45	13.61	4.50	7.11
Dic3	Dictyota tissue	24.94	0.79	0.00	0.00	1.03	15.70	7.91	3.80	8.38
Acr1	Acropora mucus	1.61	0.00	2.25	45.04	25.57	4.08	0.00	21.44	0.00
Acr2	Acropora mucus	2.28	0.00	3.09	52.11	22.91	5.26	0.00	14.35	0.00
Acr3	Acropora mucus	2.22	0.00	2.89	48.53	24.08	5.01	0.00	16.32	0.94
Dipl1	Diploria mucus	48.88	0.00	0.41	0.00	28.98	0.00	0.63	21.10	0.00
Dipl2	Diploria mucus	46.58	0.00	0.00	0.00	30.58	0.00	0.00	10.80	12.03
Dipl3	Diploria mucus	47.85	0.00	0.00	0.00	52.15	0.00	0.00	0.00	0.00
Mea1	Meandrina mucus	38.67	0.00	0.00	0.00	44.43	0.00	7.96	8.94	0.00
Mea2	Meandrina mucus	28.61	0.00	0.00	0.00	58.64	0.00	0.00	12.75	0.00
Algae1	Algae exudates	53.53	4.97	0.34	2.25	0.00	19.25	2.09	5.96	8.15
Algae2	Algae exudates	52.01	3.49	0.59	2.42	0.81	16.37	1.73	4.69	11.45
Algae3	Algae exudates	35.47	4.62	1.04	4.03	1.33	19.27	6.87	5.52	15.91
Coral1	Coral exudates	15.68	2.50	1.85	42.59	9.74	8.40	2.55	11.75	2.82
Coral2	Coral exudates	8.21	1.15	1.78	50.31	11.16	7.42	0.28	13.24	0.00
Coral3	Coral exudates	13.78	1.34	1.95	49.51	12.14	8.40	0.00	9.67	0.00
Reef	Ambient	20.11	4.28	3.01	9.47	3.89	14.31	14.45	7.31	14.43
Start1	Ambient	21.11	0.00	3.54	3.50	5.47	12.60	11.87	7.37	8.26
Start2	Ambient	18.11	0.00	3.68	8.43	5.15	15.24	11.88	6.09	13.69
Start3	Ambient	21.44	0.46	2.06	7.31	2.95	9.11	17.04	8.24	20.44

Table S6. Raw data for Fig. S3. Microbial cell counts, DOC- and nutrient concentrations.

Name	Treatment	Time	Microbes (mL^{-1})	DOC (µM)	NO2+NO3 (µM)	PO4 (µM)
A1	Algae	Start	713986	86.19	0.58	0.012
A2	Algae	Start	673311	NA	0.46	0.012
A3	Algae	Start	597027	88.06	0.51	0.012
C1	Coral	Start	645743	87.29	0.52	0.015
C2	Coral	Start	635878	NA	0.48	0.016
C3	Coral	Start	595000	87.05	0.49	0.014
Con1	Control	Start	752635	90.84	0.33	0.015
Con2	Control	Start	638041	86.89	NA	NA
Con3	Control	Start	706622	87.52	0.54	0.009
A1	Algae	6 h	807365	77.56	0.52	0.010
A2	Algae	6 h	756959	NA	0.46	0.011
A3	Algae	6 h	720878	81.86	0.58	0.013
C1	Coral	6 h	709910	80.12	0.56	0.014
C2	Coral	6 h	668694	NA	0.46	0.015
C3	Coral	6 h	733288	79.85	0.50	0.012
Con1	Control	6 h	762635	84.49	0.60	0.012
Con2	Control	6 h	721149	82.18	0.51	0.009
Con3	Control	6 h	675203	83.07	0.51	0.010
A1	Algae	12 h	916630	82.30	0.51	0.006
A2	Algae	12 h	802507	NA	0.45	0.009
A3	Algae	12 h	791630	80.59	0.60	0.009
C1	Coral	12 h	1298080	77.50	0.48	0.008
C2	Coral	12 h	823225	NA	0.44	0.011
C3	Coral	12 h	676571	79.09	0.54	0.013
Con1	Control	12 h	1256920	81.60	0.57	0.006
Con2	Control	12 h	1099529	80.94	0.58	0.015
Con3	Control	12 h	1048804	81.75	0.56	0.009
A1	Algae	1 day	1081884	79.97	0.52	0.005
A2	Algae	1 day	928261	NA	0.41	0.005
A3	Algae	1 day	1166558	80.54	0.62	0.007
C1	Coral	1 day	1174239	78.10	NA	NA
C2	Coral	1 day	1000580	NA	0.34	0.008
C3	Coral	1 day	1057283	79.73	0.37	0.010
Con1	Control	1 day	1044493	82.74	0.53	0.004
Con2	Control	1 day	1076522	79.97	0.54	0.005
Con3	Control	1 day	1044964	82.32	0.51	0.005
A1	Algae	4 days	783423	81.84	0.19	0.012
A2	Algae	4 days	827883	NA	0.07	0.011
A3	Algae	4 days	844910	83.49	0.18	0.008
C1	Coral	4 days	762568	80.43	0.19	0.009
C2	Coral	4 days	782973	NA	0.07	0.010
C3	Coral	4 days	897973	83.08	0.19	0.005
Con1	Control	4 days	773784	79.99	0.16	0.006
Con2	Control	4 days	776351	76.24	0.15	0.008
Con3	Control	4 days	808919	79.41	0.21	0.018

Table S7. Results of *DESeq2* analyses for 4 days; raw data to Fig. 5c. Only genera with significant *p* adjust are shown (out of a total of 111 genera detected after 4 days).

Family_Genus	baseMean	log2FoldChange	lfcSE	stat	*p*-value	*p* adj
Phycisphaeraceae_Urania-1B-19_m.s.g.	37.50	2.10	0.45	4.63	0.0000	0.0001
Flavobacteriales_unclassified	20.57	1.88	0.48	3.91	0.0001	0.0026
Rhodobacteraceae_Shimia	38.43	1.71	0.48	3.60	0.0003	0.0059
Saprospiraceae_uncultured	20.29	1.52	0.54	2.83	0.0047	0.0377
Vibrionaceae_unclassified	19.04	1.34	0.50	2.68	0.0075	0.0488
Ectothiorhodospiraceae_uncultured	39.71	1.30	0.38	3.44	0.0006	0.0091
Phycisphaeraceae_CL500-3	73.65	1.25	0.42	2.98	0.0029	0.0294
Thiotrichaceae_uncultured	29.93	1.24	0.40	3.11	0.0019	0.0207
Rhodobacteraceae_unclassified	589.73	1.14	0.22	5.29	0.0000	0.0000
OM190_ge	110.63	0.87	0.24	3.64	0.0003	0.0059
Alphaproteobacteria_uncultured_ge	48.73	-1.20	0.44	-2.73	0.0063	0.0449
Saccharospirillaceae_Oleibacter	32.11	-1.49	0.53	-2.82	0.0048	0.0377
Puniceicoccaceae_MB11C04_m.g.	13.47	-1.68	0.57	-2.92	0.0035	0.0323
Methylophilaceae_OM43_clade	14.58	-2.37	0.75	-3.16	0.0016	0.0196
Marine_Group_III_ge	5.81	-3.78	1.39	-2.72	0.0065	0.0449
Methylophagaceae_Marine_Methylotrophic_g_3	128.28	-4.20	1.29	-3.25	0.0012	0.0162
Methylophagaceae_unclassified	188.58	-5.40	1.14	-4.73	0.0000	0.0001

Table S8. Raw data to Fig. 6a. Predicted pathway class abundance (MicFunPred).

Treatment	Time	ID	Energy Metabolism	Amino Acids	Carbo-hydrates	Fatty Acids and Lipids	Secondary metabolites
Algae	Start	A1	22418	16161	12280	7283	6275
Algae	Start	A2	24080	16505	13337	7542	6797
Algae	Start	A3	17407	6183	4745	6091	4099
Coral	Start	C1	21875	15384	12107	6904	6228
Coral	Start	C2	22273	14722	12049	8598	6196
Coral	Start	C3	21520	15854	11824	7032	6049
Control	Start	Con1	23357	14893	12714	7176	6534
Control	Start	Con2	21502	14485	11824	6774	6033
Control	Start	Con3	22830	15331	12395	7037	6310
Algae	6 h	A1	20381	13707	11532	6878	5625
Algae	6 h	A2	21169	14016	11793	6889	5830
Algae	6 h	A3	20428	14200	11201	7830	5570
Coral	6 h	C1	21431	13220	8224	7832	4824
Coral	6 h	C2	20647	12673	8224	7372	4648
Coral	6 h	C3	19755	11293	10952	6691	4810
Control	6 h	Con1	19188	13575	10605	7771	5423
Control	6 h	Con2	17746	11509	6943	6603	4095
Control	6 h	Con3	20571	14180	11565	6902	5659
Algae	12 h	A1	22081	13772	6762	9164	4978
Algae	12 h	A2	29227	18064	16758	10997	7140
Algae	12 h	A3	27705	18324	8497	12048	6305
Coral	12 h	C1	38139	29902	21315	16766	10232
Coral	12 h	C2	32902	27765	18184	15542	11611
Coral	12 h	C3	29686	21593	12218	12718	6672
Control	12 h	Con1	22550	13381	6786	9512	5108
Control	12 h	Con2	23941	16870	9744	9947	5403
Control	12 h	Con3	25483	17648	7853	11136	5842
Algae	1 day	A1	59208	54291	23458	32083	16586
Algae	1 day	A2	55333	47963	25357	28216	13175
Algae	1 day	A3	58839	53391	27857	31068	15787
Coral	1 day	C1	53193	44657	19633	28543	14082
Coral	1 day	C2	68107	63784	41621	38413	19439
Coral	1 day	C3	62118	57683	40751	30384	18282
Control	1 day	Con1	64309	61930	44629	38378	19007
Control	1 day	Con2	62822	62119	45274	33379	19152
Control	1 day	Con3	59046	52004	23821	33019	17238
Algae	4 days	A1	69145	54011	24819	37645	16050
Algae	4 days	A2	72811	55480	25648	38644	16116
Algae	4 days	A3	75085	57666	27013	38131	18389
Coral	4 days	C1	90982	81437	56127	42236	26523
Coral	4 days	C2	92795	80717	55062	48205	25573
Coral	4 days	C3	88055	85263	53059	50319	31733
Control	4 days	Con1	68873	54646	25517	37476	16597
Control	4 days	Con2	70014	61847	25749	38811	16857
Control	4 days	Con3	72704	55836	26483	36924	18049

Supplementary References

1. Vidal-Melgosa S, Sichert A, Francis TB, Bartosik D, Niggemann J, Wichels A, Willats WGT, Fuchs BM, Teeling H, Becher D, Schweder T, Amann R, Hehemann J-H. 2021. Diatom fucan polysaccharide precipitates carbon during algal blooms. 1. Nature Communications 12:1150.
2. Apprill A, Weber LG, Santoro AE. 2016. Distinguishing between microbial habitats unravels ecological complexity in coral microbiomes. mSystems 1:e00143-16.
3. Lima LFO, Alker AT, Papudeshi B, Morris MM, Edwards RA, de Putron SJ, Dinsdale EA. 2023. Coral and seawater metagenomes reveal key microbial functions to coral health and ecosystem functioning shaped at reef scale. Microb Ecol 86:392–407.
4. Marchioro GM, Glasl B, Engelen AH, Serrão EA, Bourne DG, Webster NS, Frade PR. 2020. Microbiome dynamics in the tissue and mucus of acroporid corals differ in relation to host and environmental parameters. PeerJ 8:e9644.

5. Zou Y, Chen Y, Wang L, Zhang S, Li J. 2022. Differential responses of bacterial communities in coral tissue and mucus to bleaching. Coral Reefs 41:951–960.

6. Nelson CE, Goldberg SJ, Wegley Kelly L, Haas AF, Smith JE, Rohwer F, Carlson CA. 2013. Coral and macroalgal exudates vary in neutral sugar composition and differentially enrich reef bacterioplankton lineages. ISME J 7:962–979.

7. Taniguchi A, Kuroyanagi Y, Aoki R, Eguchi M. 2023. Community structure and predicted functions of actively growing bacteria responsive to released coral mucus in surrounding seawater. Microbes and Environments 38:ME23024.

8. Taniguchi A, Yoshida T, Hibino K, Eguchi M. 2015. Community structures of actively growing bacteria stimulated by coral mucus. Journal of Experimental Marine Biology and Ecology 469:105–112.

9. Weber L, Soule MK, Longnecker K, Becker CC, Huntley N, Kujawinski EB, Apprill A. 2022. Benthic exometabolites and their ecological significance on threatened Caribbean coral reefs. 1. ISME COMMUN 2:1–13.

10. Bourne DG, van der Zee MJJ, Botté ES, Sato Y. 2013. Sulfur-oxidizing bacterial populations within cyanobacterial dominated coral disease lesions. Environmental Microbiology Reports 5:518–524.

11. McDevitt-Irwin JM, Baum JK, Garren M, Vega Thurber RL. 2017. Responses of coral-associated bacterial communities to local and global stressors. Front Mar Sci 4:262.

12. Meyer JL, Castellanos-Gell J, Aeby GS, Häse CC, Ushijima B, Paul VJ. 2019. Microbial community shifts associated with the ongoing stony coral tissue loss disease outbreak on the Florida reef tract. Frontiers in Microbiology 10.

13. Lee STM, Davy SK, Tang S-L, Fan T-Y, Kench PS. 2015. Successive shifts in the microbial community of the surface mucus layer and tissues of the coral *Acropora muricata* under thermal stress. FEMS Microbiology Ecology 91:fiv142.

14. Allers E, Niesner C, Wild C, Pernthaler J. 2008. Microbes enriched in seawater after addition of coral mucus. Appl Environ Microbiol 74:3274–3278.

15. Gignoux-Wolfsohn SA, Vollmer SV. 2015. Identification of candidate coral pathogens on white band disease-infected staghorn coral. PLOS ONE 10:e0134416.

16. Lee STM, Davy SK, Tang S-L, Kench PS. 2016. Mucus sugar content shapes the bacterial community structure in thermally stressed *Acropora muricata*. Front Microbiol 7:371.

17. Heitzman JM, Caputo N, Yang S-Y, Harvey BP, Agostini S. 2022. Recurrent disease outbreak in a warm temperate marginal coral community. Marine Pollution Bulletin 182:113954.

18. Sun H, Zheng H, Jiang Y, Liang J, Liao B, Wang R, Li A, Xiao B. 2022. Elevated temperature alters bacterial community composition and metabolism in seawaters of coral reef ecosystem: An evidence of laboratory experiment with Acropora digitifera bleaching. Ecological Indicators 139:108886.

19. Li J, Long L, Zou Y, Zhang S. 2021. Microbial community and transcriptional responses to increased temperatures in coral *Pocillopora damicornis* holobiont. Environmental Microbiology 23:826–843.

20. Ziegler M, Seneca FO, Yum LK, Palumbi SR, Voolstra CR. 2017. Bacterial community dynamics are linked to patterns of coral heat tolerance. Nat Commun 8:14213.
